# Supplementary figures and images for: Key residues in the VDAC2-BAK complex can be targeted to modulate apoptosis
Source: PLoS Biol. 2024 May 2;22(5):e3002617. doi: 10.1371/journal.pbio.3002617 (PMC11098506; doi:10.1371/journal.pbio.3002617)

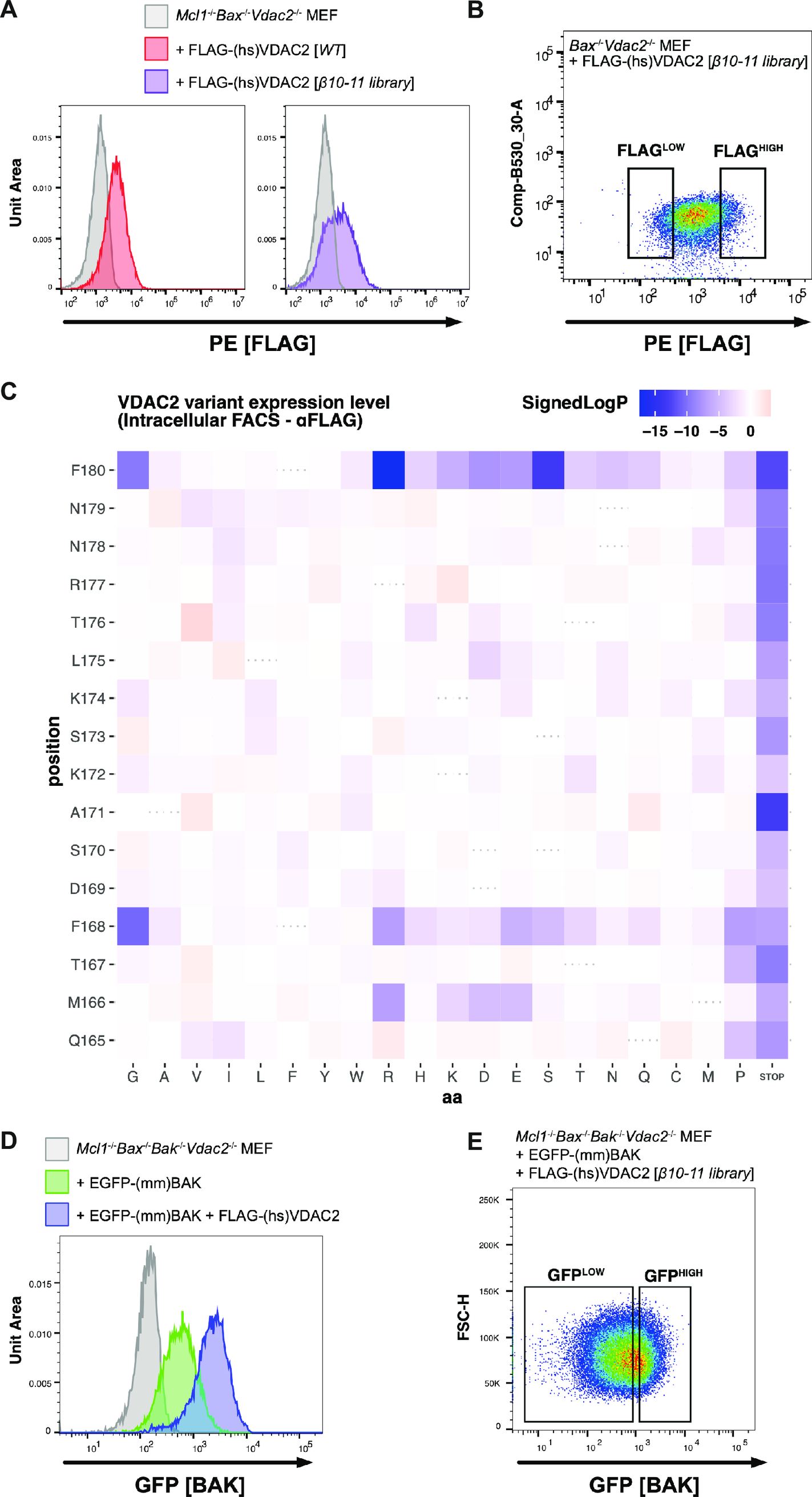

Supplement: S1 Fig — (A) MCL1/BAX/VDAC2-deficient MEFs engineered by retroviral infection to express wild-type FLAG-VDAC2 or a library of uniquely barcoded FLAG-tagged VDAC2 substitution variants within the β10–11 loop were fixed and stained with PE-conjugated anti-FLAG antibody. The distribution of expression observed in cells expressing VDAC2 variants from the library was broader than for wild-type VDAC2, reflecting variation in expression level for clones within the library. (B) BAX/VDAC2-deficient MEFs expressing barcoded FLAG-VDAC2 substitution variants were fixed and stained with PE-conjugated anti-FLAG antibody. FLAGhigh and FLAGlow populations were sorted and used to generate Illumina sequencing libraries in order to identify substitutions that impair or enhance VDAC2 expression. (C) Heatmap representation of DMS screen to identify residues within hsVDAC2 β10–11 that influence VDAC2 expression level. Illumina sequencing was performed on the sorted FLAGhigh and FLAGlow populations to quantitate PCR-amplified barcode levels. Mann–Whitney p-values were calculated comparing the log2-fold differences for barcodes associated with each coding substitution relative to the barcodes associated with wild-type VDAC2 coding sequence. Data are represented as signed log transformed p-values to reflect the direction of change: negative/blue for variants skewed towards the FLAGlow fraction; positive/red for variants skewed towards the FLAGhigh fraction. (D) MCL1/BAX/BAK/VDAC2-deficient MEFs were engineered by retroviral infection to express GFP-tagged mouse BAK. In the absence of VDAC2, the expression levels of GFP-BAK are low (green histogram). When FLAG-tagged wild-type human VDAC2 is expressed in these cells, the GFP-fluorescent signal is markedly enhanced (blue histogram). (E) MCL1/BAX/BAK/VDAC2-deficient MEFs expressing uniquely barcoded FLAG-tagged VDAC2 substitution variants within the β10–11 loop were sorted into GFPhigh and GFPlow fractions. These were used to generate Illumina sequenci [file pbio.3002617.s001.tif]

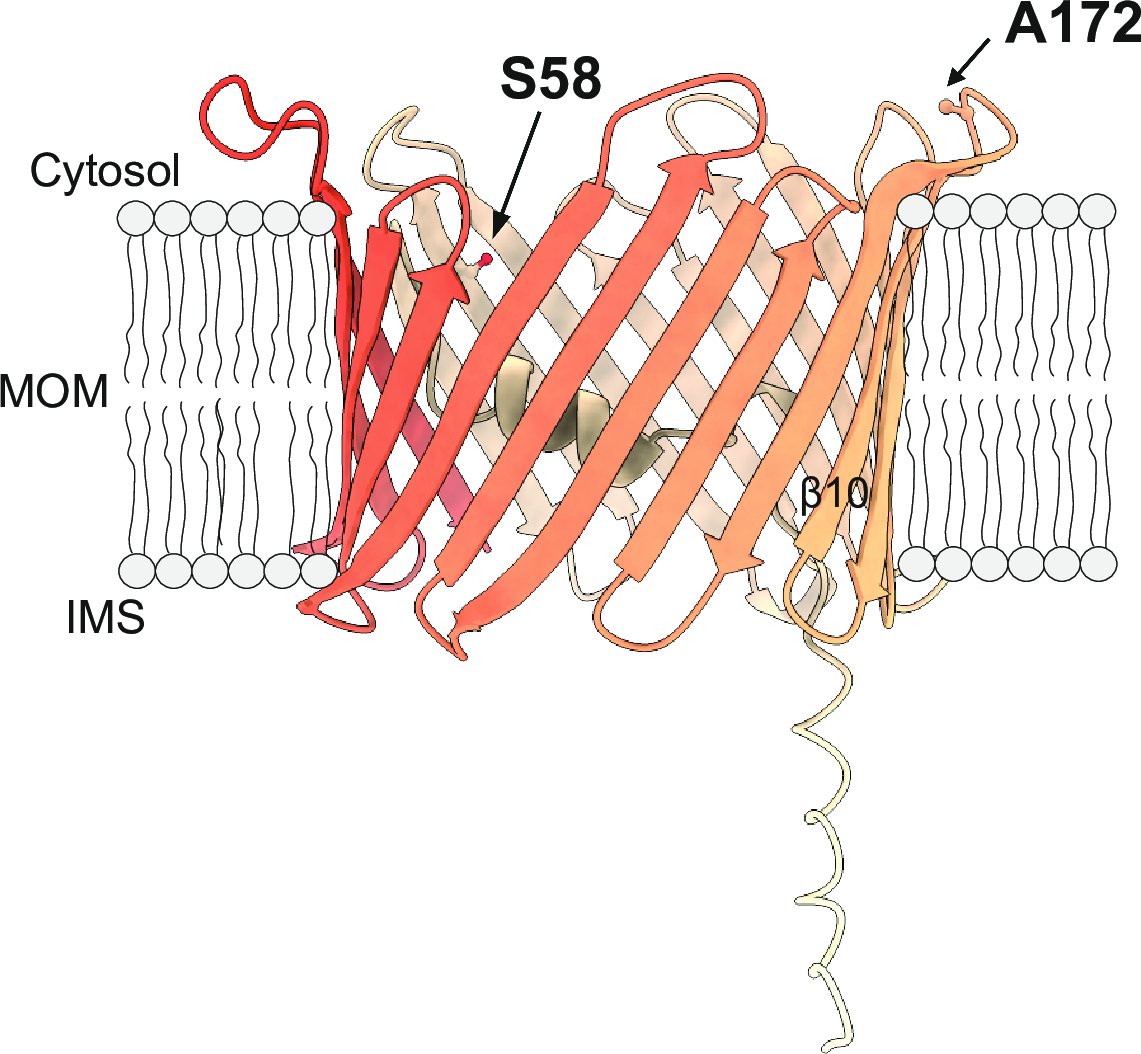

Supplement: S2 Fig — Relates to Fig 2. (TIF) [file pbio.3002617.s002.tif]

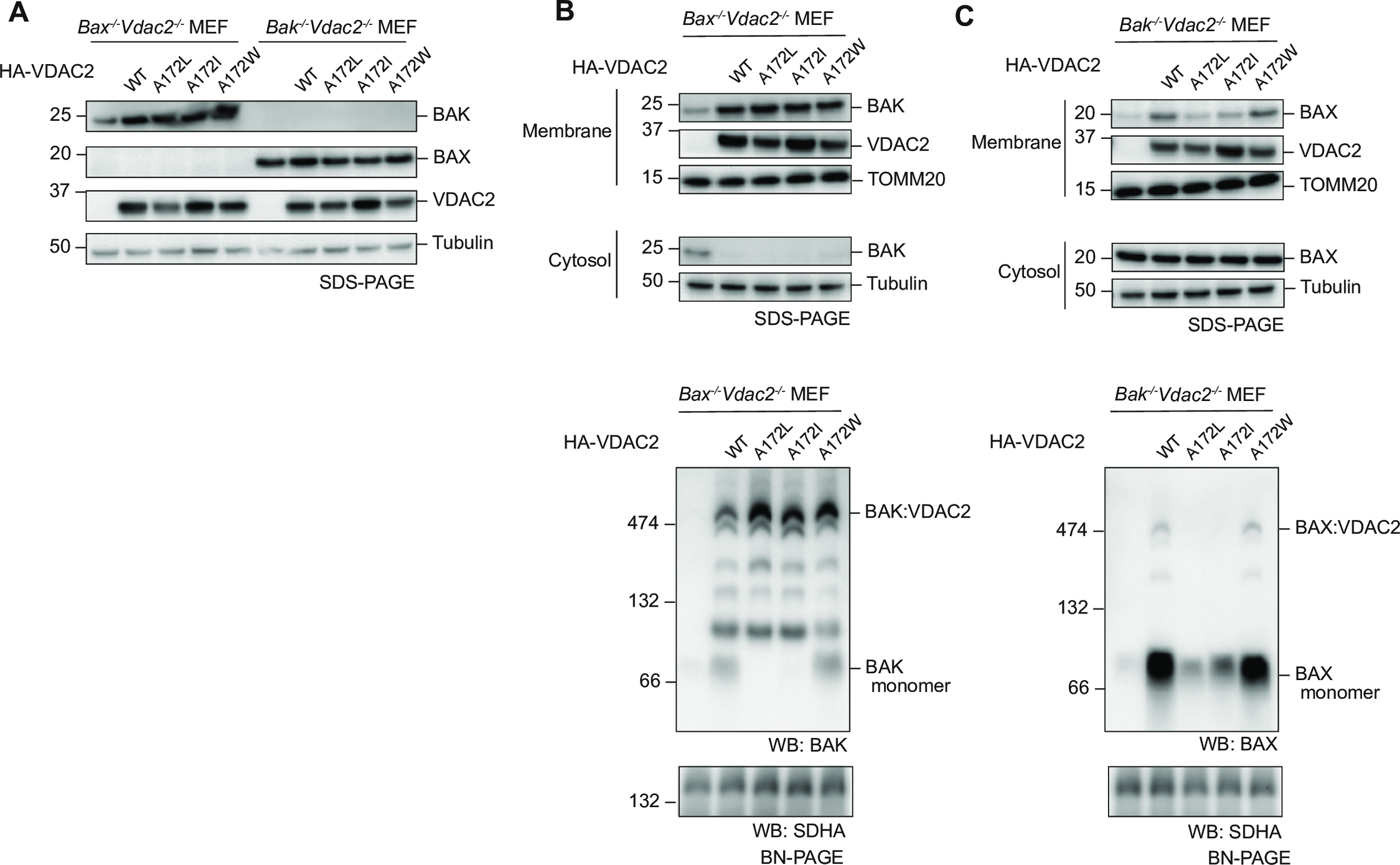

Supplement: S3 Fig — (TIF) [file pbio.3002617.s003.tif]

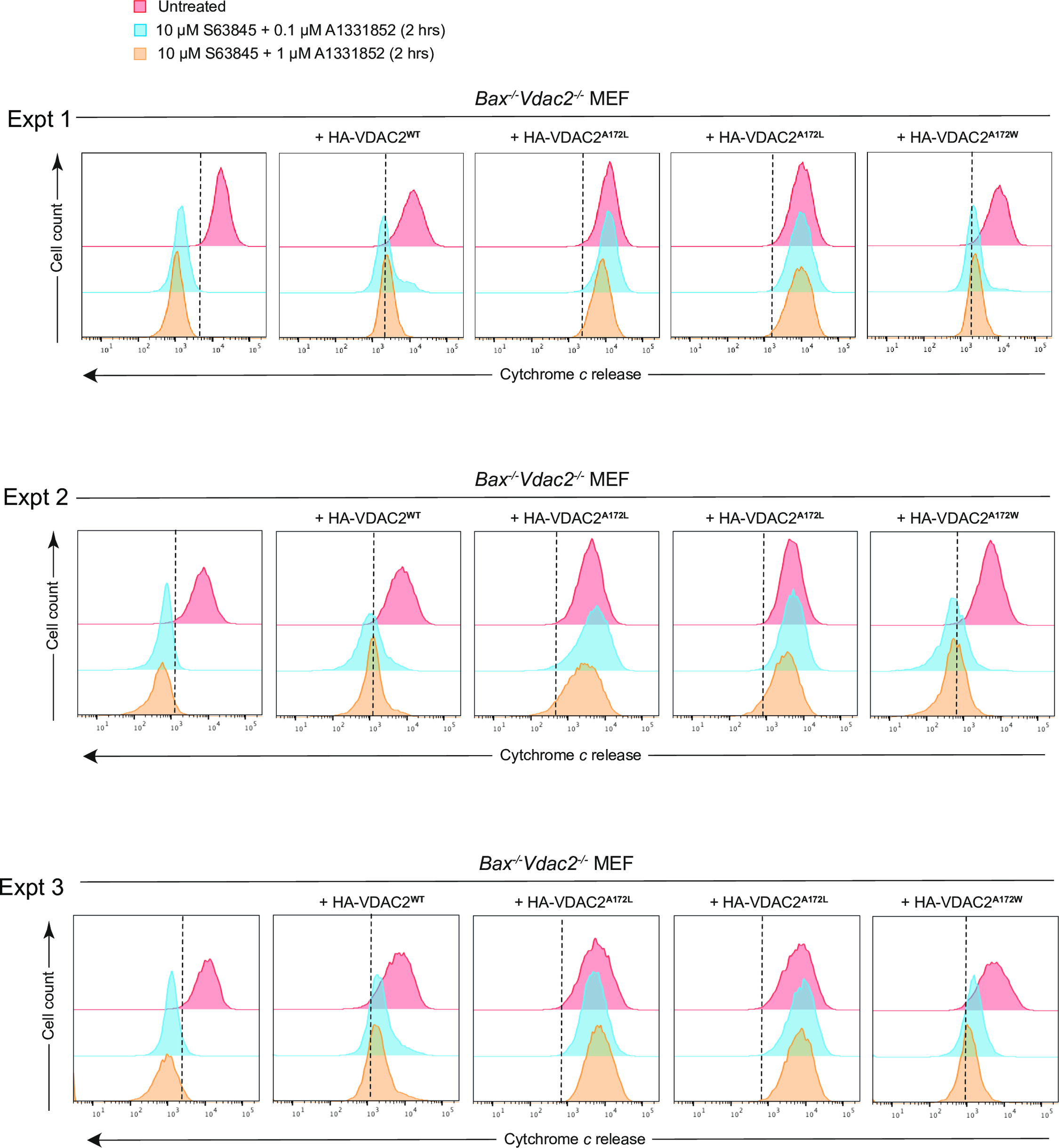

Supplement: S4 Fig — (TIF) [file pbio.3002617.s004.tif]

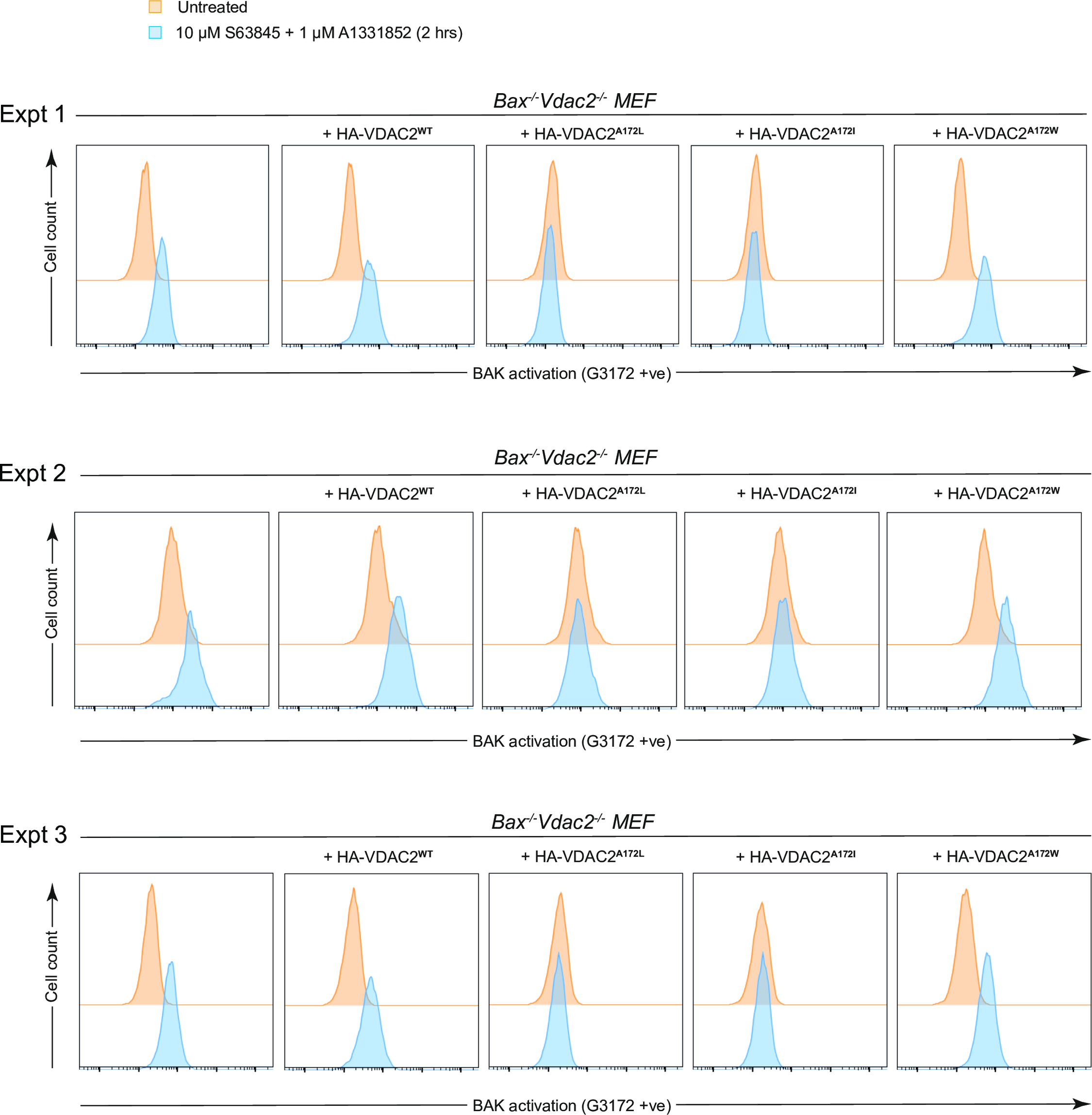

Supplement: S5 Fig — (TIF) [file pbio.3002617.s005.tif]
