# Supplementary material for: Key residues in the VDAC2-BAK complex can be targeted to modulate apoptosis
Source: PLoS Biol. 2024 May 2;22(5):e3002617. doi: 10.1371/journal.pbio.3002617 (PMC11098506; doi:10.1371/journal.pbio.3002617)
Supplement: S1 Raw Images — (PDF) [file pbio.3002617.s008.pdf]

Fig. 2C top

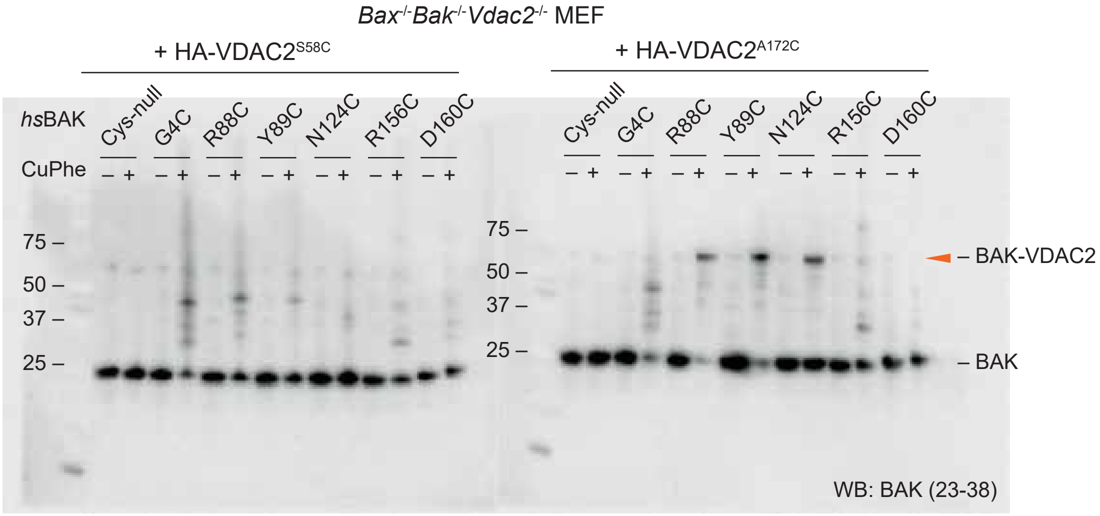

Fig. 2C bottom

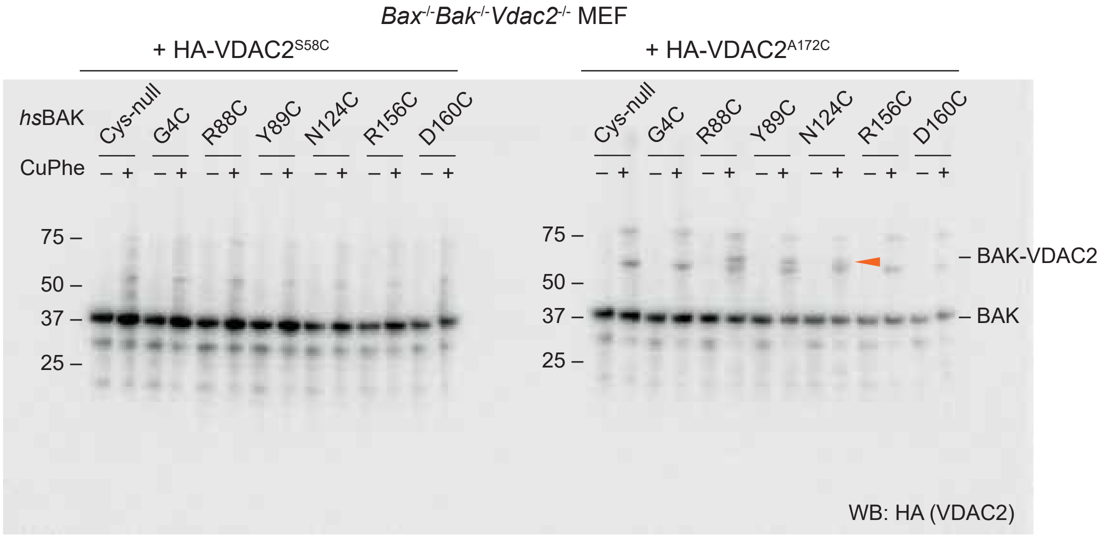

Fig. 2D top

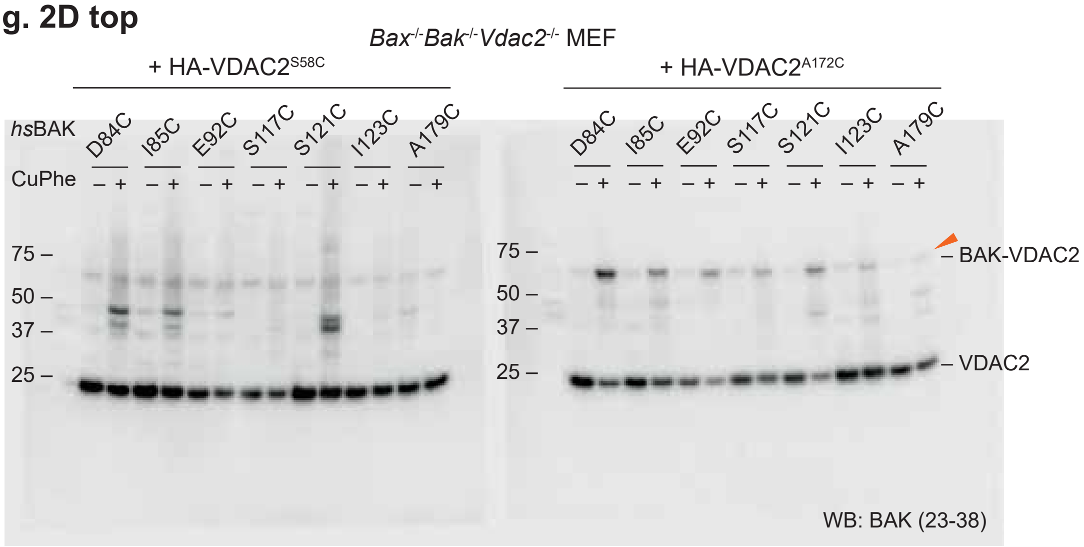

Fig. 2D bottom

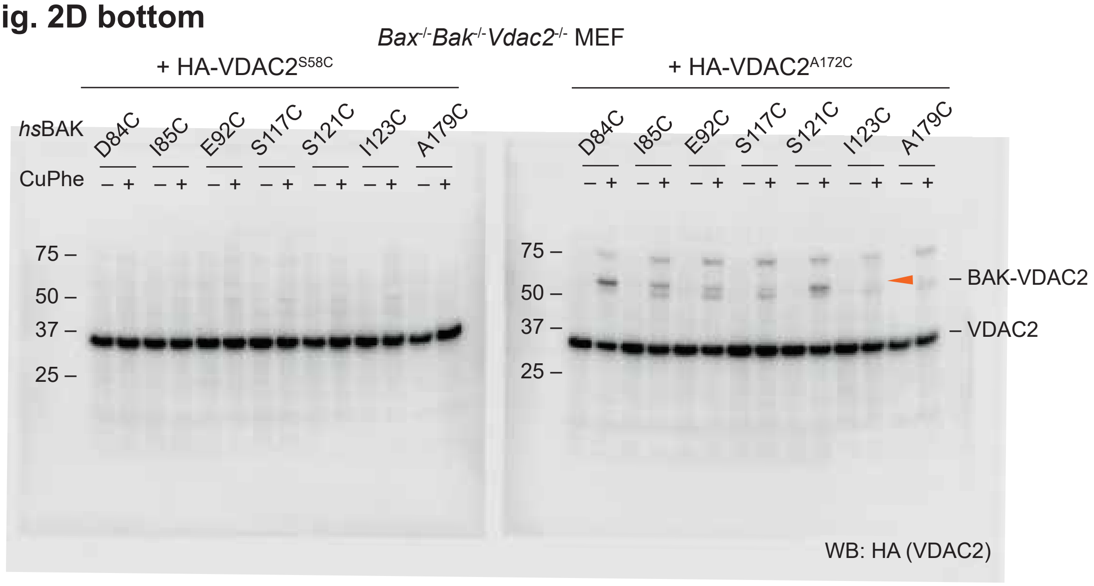

Fig. 4A

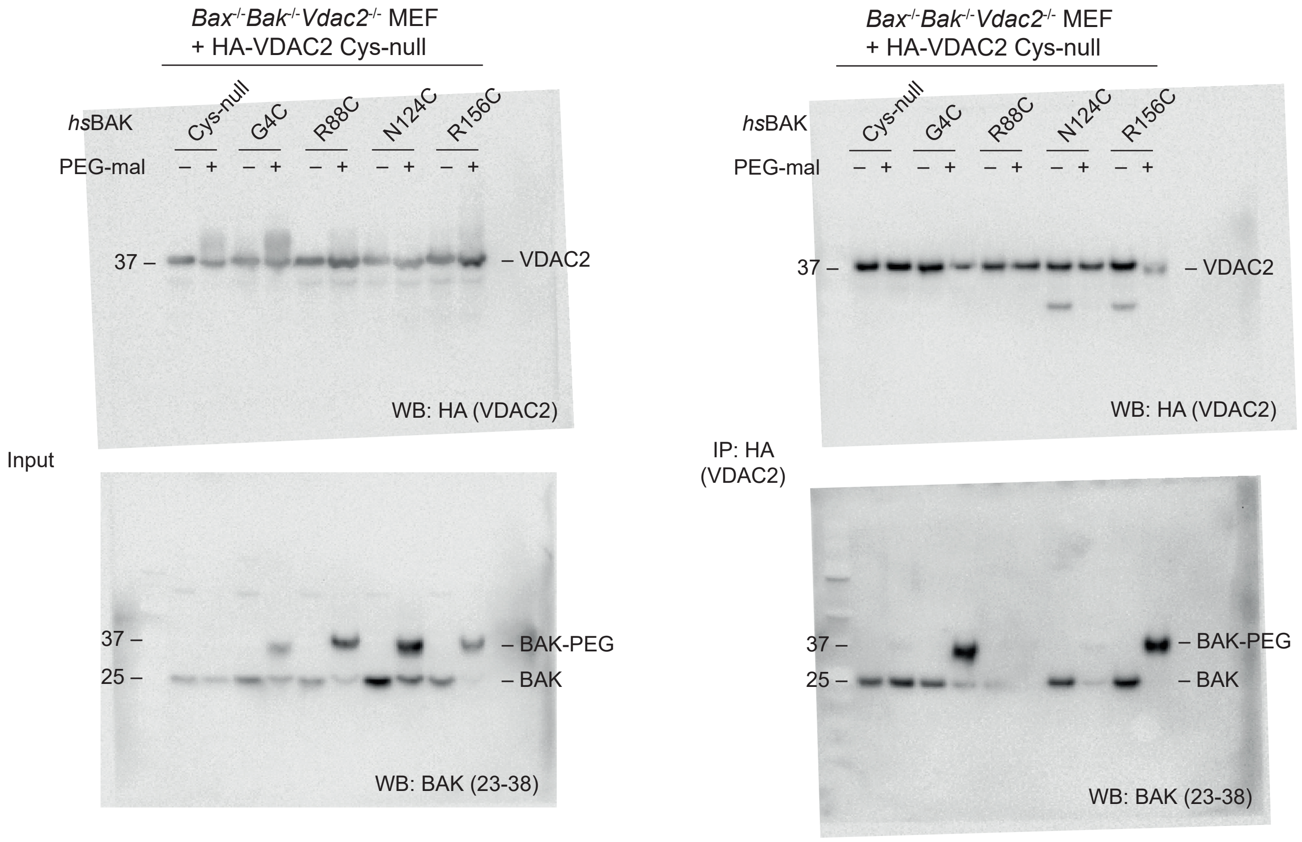

Fig. 4B

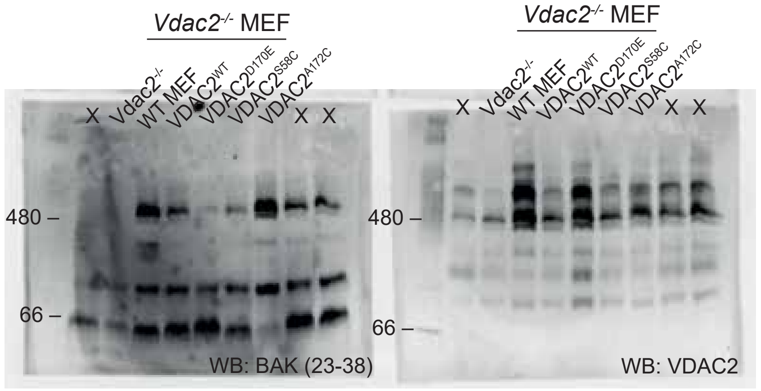

Fig. 4C

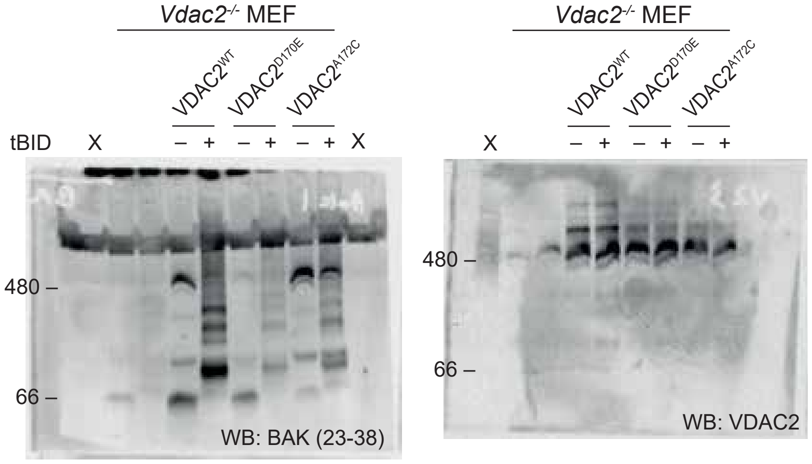

Fig. 4D

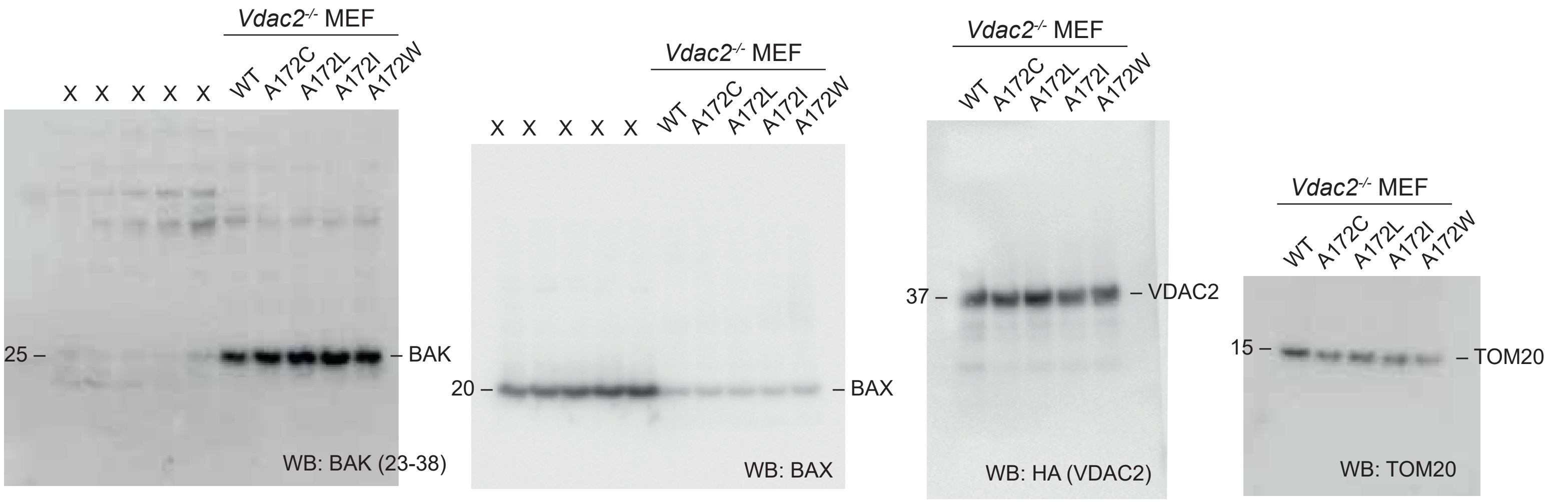

Fig. 4E

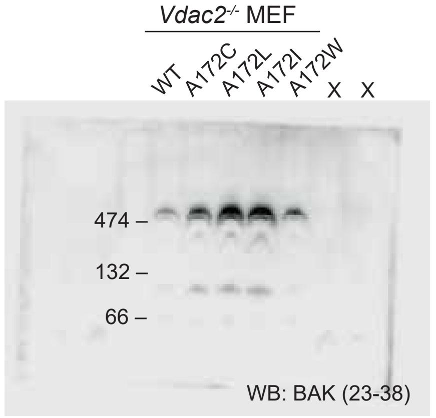

Fig. 4F

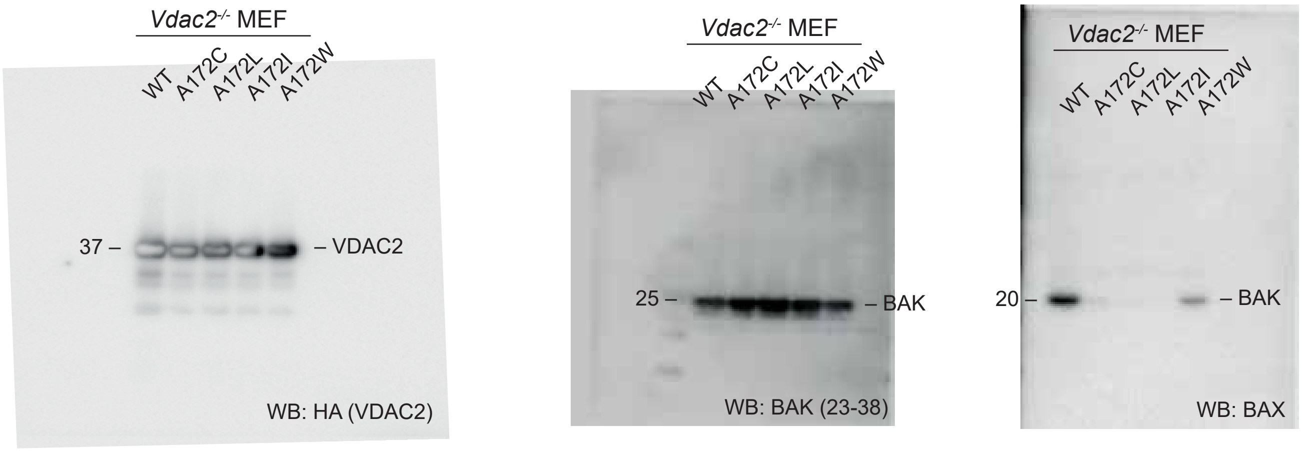

Fig. 4G

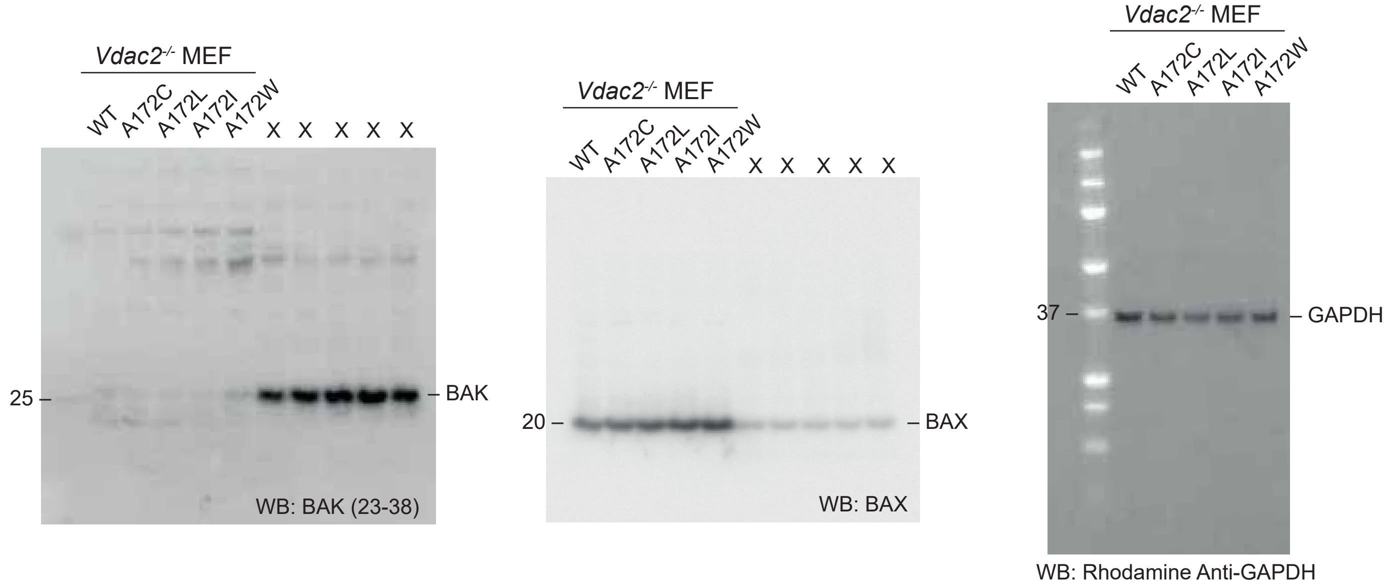

Fig. 4H

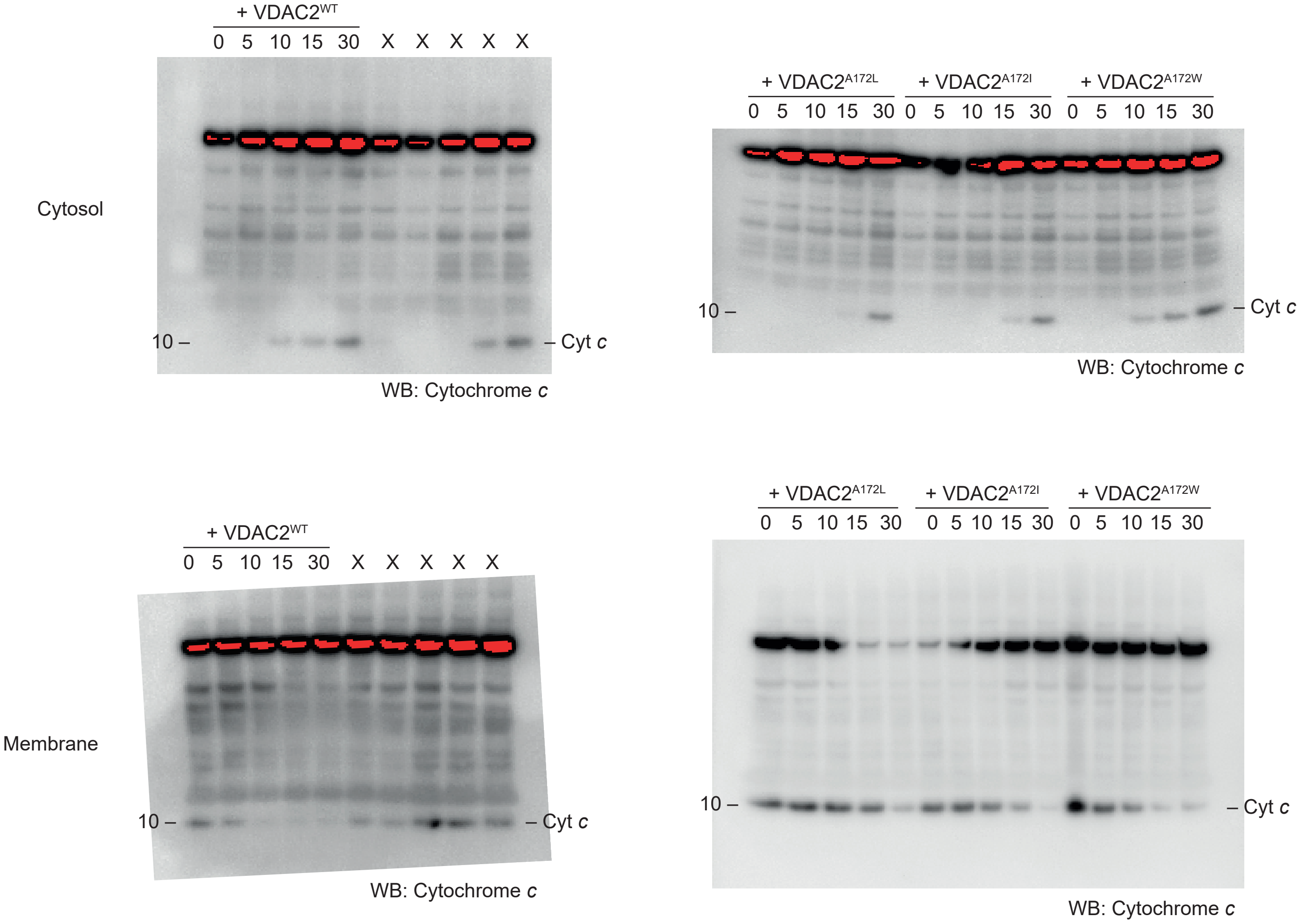
+ VDAC2WT
0 5 10 15 30 X X X X X
Cytosol
10 –
– Cyt c
WB: Cytochrome c
+ VDAC2A172L
+ VDAC2A172I
+ VDAC2A172W
0 5 10 15 30 0 5 10 15 30 0 5 10 15 30
10 –
– Cyt c
WB: Cytochrome c
+ VDAC2WT
0 5 10 15 30 X X X X X
Membrane
10 –
– Cyt c
WB: Cytochrome c
+ VDAC2A172L
+ VDAC2A172I
+ VDAC2A172W
0 5 10 15 30 0 5 10 15 30 0 5 10 15 30
10 –
– Cyt c
WB: Cytochrome c

Fig. S1A

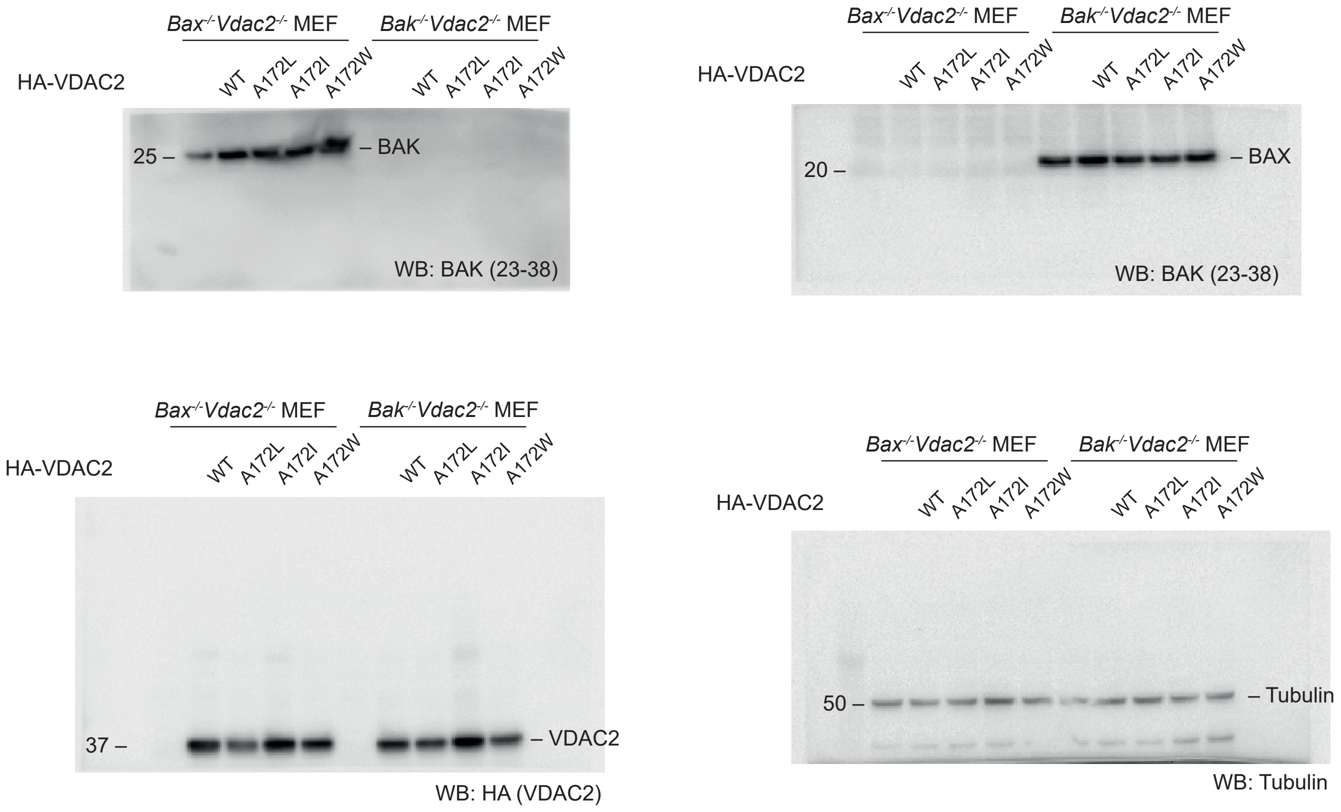
Bax-/-Vdac2-/- MEF
Bak-/-Vdac2-/- MEF
HA-VDAC2
WT
A172L
A172I
A172W
25 –
– BAK
WB: BAK (23-38)
20 –
– BAX
WB: BAK (23-38)
37 –
– VDAC2
WB: HA (VDAC2)
50 –
– Tubulin
WB: Tubulin

Fig. S1B

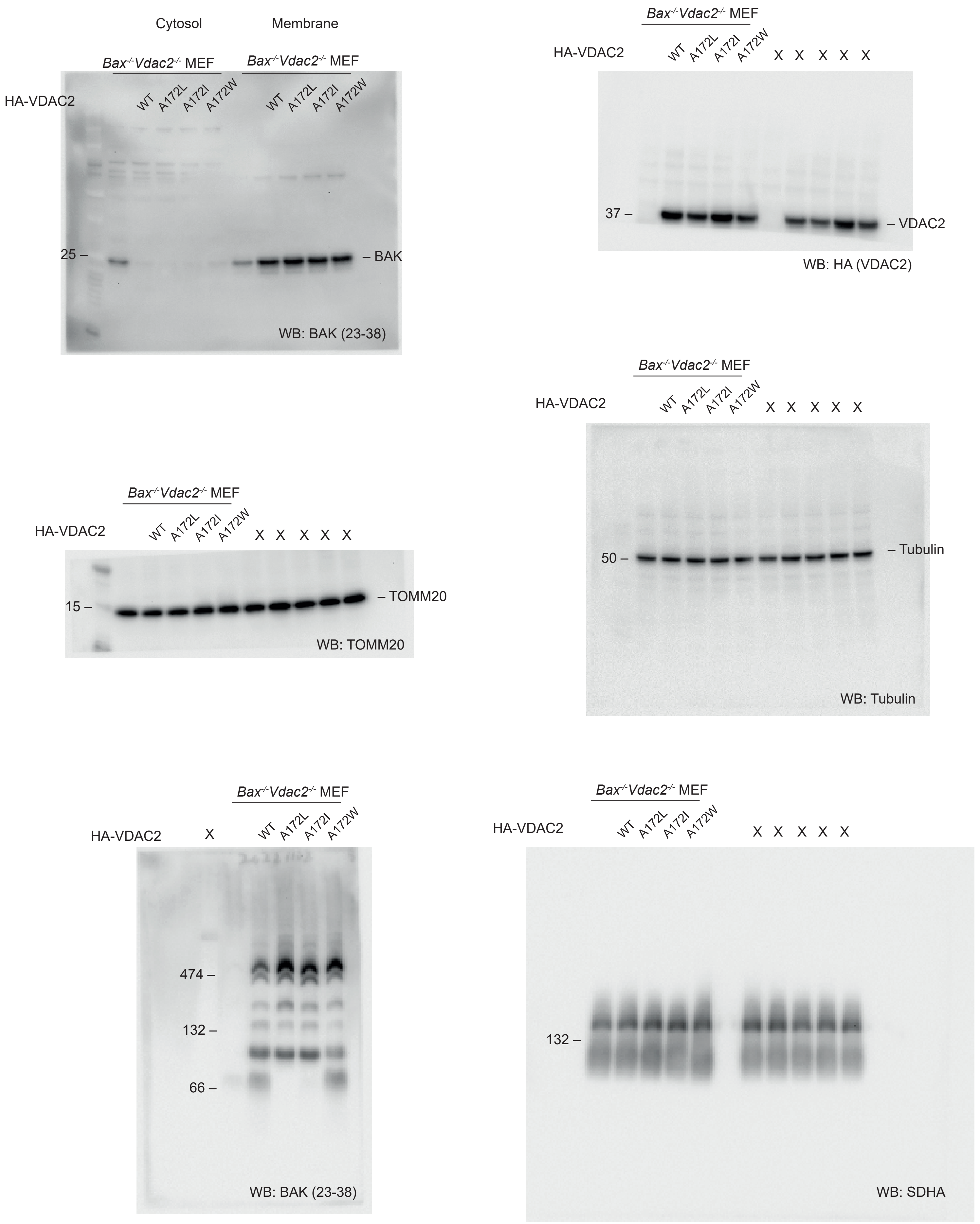
Cytosol
Membrane
Bax-/-Vdac2-/- MEF
Bax-/-Vdac2-/- MEF
HA-VDAC2
WT
A172L
A172I
A172W
WT
A172L
A172I
A172W
25 –
– BAK
WB: BAK (23-38)
Bax-/-Vdac2-/- MEF
HA-VDAC2
WT
A172L
A172I
A172W
X X X X X
37 –
– VDAC2
WB: HA (VDAC2)
Bax-/-Vdac2-/- MEF
HA-VDAC2
WT
A172L
A172I
A172W
X X X X X
50 –
– Tubulin
WB: Tubulin
Bax-/-Vdac2-/- MEF
HA-VDAC2
WT
A172L
A172I
A172W
X X X X X
15 –
– TOMM20
WB: TOMM20
Bax-/-Vdac2-/- MEF
HA-VDAC2
X
WT
A172L
A172I
A172W
474 –
132 –
66 –
WB: BAK (23-38)
Bax-/-Vdac2-/- MEF
HA-VDAC2
WT
A172L
A172I
A172W
X X X X X
132 –
WB: SDHA

Fig. S1C

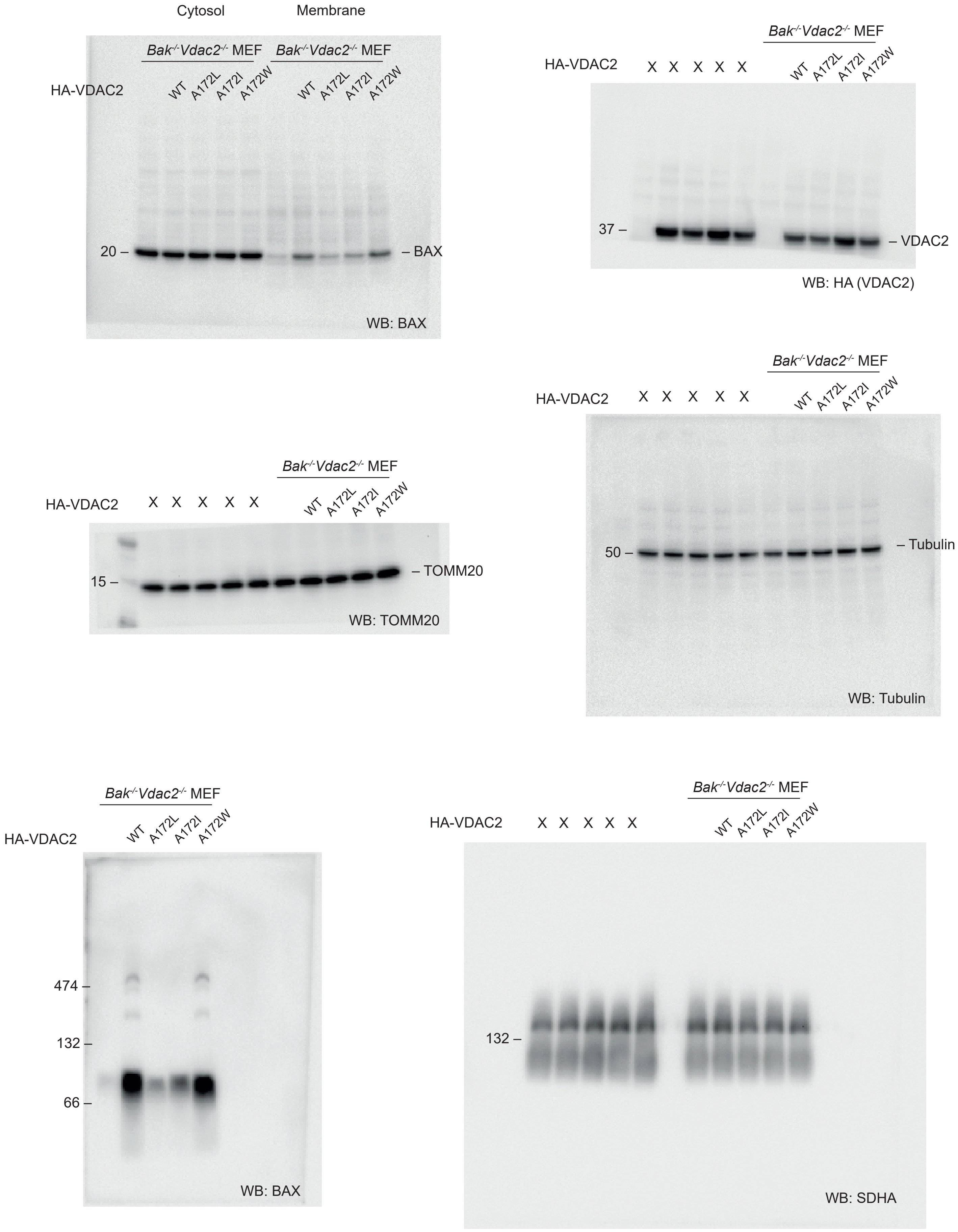
Cytosol
Membrane
$Bak^{-/-}Vdac2^{-/-}$ MEF
$Bak^{-/-}Vdac2^{-/-}$ MEF
HA-VDAC2
WT A172L A172I A172W
WT A172L A172I A172W
20 –
– BAX
WB: BAX
$Bak^{-/-}Vdac2^{-/-}$ MEF
HA-VDAC2
X X X X X
WT A172L A172I A172W
37 –
– VDAC2
WB: HA (VDAC2)
$Bak^{-/-}Vdac2^{-/-}$ MEF
HA-VDAC2
X X X X X
WT A172L A172I A172W
15 –
– TOMM20
WB: TOMM20
$Bak^{-/-}Vdac2^{-/-}$ MEF
HA-VDAC2
X X X X X
WT A172L A172I A172W
50 –
– Tubulin
WB: Tubulin
$Bak^{-/-}Vdac2^{-/-}$ MEF
HA-VDAC2
WT A172L A172I A172W
474 –
132 –
66 –
WB: BAX
$Bak^{-/-}Vdac2^{-/-}$ MEF
HA-VDAC2
X X X X X
WT A172L A172I A172W
132 –
WB: SDHA
