# Supplementary material for: Key residues in the VDAC2-BAK complex can be targeted to modulate apoptosis
Source: PLoS Biol. 2024 May 2;22(5):e3002617. doi: 10.1371/journal.pbio.3002617 (PMC11098506; doi:10.1371/journal.pbio.3002617)
Supplement: S1 Methods — (DOCX) [file pbio.3002617.s009.docx]

**Appendix Supplementary Methods**

**Contents**

BAK (α1–α9):mVDAC2 Docking Page 1

Molecular Dynamics Simulation Page 2

References Page 2

**BAK (α1–α9):mVDAC2 Docking**

Molecular docking of BAK to membrane-bound mVDAC2 was conducted initially utilizing a model of the BAK α9 (I188–S211) domain. The BAK α9 peptide was created by aligning against an 18-residue poly-alanine helical peptide, created using the UCSF Chimera program [1] enforcing α-helical secondary structure restraints. Docking of the peptide made use of the integrative LightDock utility [2]. LightDock uses an artificial-intelligence ‘glow-worm’ swarm-based algorithm to identify initial positions of the docked ‘ligand’ in reference to the ‘receptor’, followed by sampling of flexible backbone orientations with the anisotropic network model (ANM) and energy minimization based on a Glowworm Swarm Optimization (GSO) algorithm. The AlphaFold structure of mVDAC2 (M1–A295; UniProt Q60939) was embedded within a mimicked MOM using the CHARMM-GUI webserver [3,4].

Docking of the BAK α9 peptide was restrained to interact only with mVDAC2 β7–β11 (residues K121–R186), generating 101 swarm centres with simulations produced using the default parameters. After clustering redundant solutions, a total of 17 models were orientated with the long axis of the α9 peptide approximately parallel to the *z*-axis of mVDAC2, and the N-terminus of the peptide orientated towards the extracellular region of the membrane. Using the top 10 solutions (assessed using the LightDock fastdfire scoring function) of this BAK α9:mVDAC2 complex, a second round of docking was conducted using a minimised BAK α1-8 model absent α9 (residues L19–L181). The top 10 results from each of these 10 docking runs (a total of 100 solutions) was reduced based on criteria that satisfied both the cross-linking data (BAK D84, R88 and S121 within 5 Å of mVDAC2 residue A121) and that permitted joining of the two BAK α1-8 and α9 domains. The MODELLER v9 utility [5] was used to build the BAK α8-9 loop, connecting the BAK α1-8 and α9 domains, forming a near full-length BAK(α1–α9):VDAC2 complex. The position of membrane phosphate head-groups allowed for the identification and definition of the boundary of the membrane along the *z*-axis, ensuring the secondary docked poses of BAK α1–α8 explored regions of mVDAC2 that were solvent exposed and not within the apolar membrane region.

**Molecular Dynamics Simulation**

Following initial construction of the BAK(α1–α9):VDAC2 complexes, they were subsequently subjected to molecular dynamics simulations using the Gromacs (v2021)[6] suite with the Charmm36m force field [7], making use of the CHARMM-GUI webserver [3,4] for membrane integration. The components of the membrane followed previous work of BAK core-dimers modelled within the same environment [8]. Sodium and chloride ions were included to neutralize the system and attain an ionic strength of 0.1 M. The temperature of the protein, solvent and membrane was coupled independently using the Nose-Hoover thermostat applying a coupling time of 1 ps at 310 K. The pressure of the system was maintained against a reference of 1 bar using semi-isotropic pressure coupling with the Parrinello-Rahman barostat, [9] using a coupling time of 5.0 ps. van der Waals interactions used a cut-off of 12 Å, and the force-switch modifier to ensure the potential is zero at the cut-off distance. The particle-mesh Ewald method [10] was used for electrostatic interactions within a similar cut-off of 12 Å. Neighbour searching made use of the Verlet grid cut-off scheme, update every 100 steps (following usual practice when using GPU hardware for the calculations). Periodic boundary conditions were applied in all directions. Bonds to hydrogen atoms were constrained with the LINCS algorithm, [11] allowing an integration time step of 2 fs. Initial minimization and equilibration followed established procedures outlined by the CHARMM-GUI via a steepest descent protocol, followed by short positionally restrained equilibration in the NVT and NPT ensemble. Simulations were then allowed to progress in the NPT ensemble for 500-ns production calculations. A total of 38 complexes were successfully sampled, generating an aggregate of 19 µs.

**References**

1. Pettersen, E. F., Goddard, T. D., Huang, C. C., Couch, G. S., Greenblatt, D. M., Meng, E. C., & Ferrin, T. E. (2004). UCSF Chimera—A Visualization System for Exploratory Research and Analysis. *J Comput Chem*, *25*, 1605–1612. https://doi.org/10.1002/jcc.20084

2. Roel-Touris, J., Jiménez-García, B., & Bonvin, A. M. J. J. (2020). Integrative modeling of membrane-associated protein assemblies. *Nature Communications*, *11*(1), 1–11. https://doi.org/10.1038/s41467-020-20076-5

3. Jo, S., Kim, T., Iyer, V. G., & Im, W. (2008). CHARMM-GUI: A web-based graphical user interface for CHARMM. *Journal of Computational Chemistry*, *29*(11), 1859–1865. https://doi.org/10.1002/jcc.20945

4. Lee, J., Cheng, X., Swails, J. M., Yeom, M. S., Eastman, P. K., Lemkul, J. A., Wei, S., Buckner, J., Jeong, J. C., Qi, Y., Jo, S., Pande, V. S., Case, D. A., Brooks, C. L., MacKerell, A. D., Klauda, J. B., & Im, W. (2016). CHARMM-GUI Input Generator for NAMD, GROMACS, AMBER, OpenMM, and CHARMM/OpenMM Simulations Using the CHARMM36 Additive Force Field. *Journal of Chemical Theory and Computation*, *12*(1), 405–413.

[https://doi.org/10.1021/acs.jctc.5b00935](https://doi.org/10.1021/acs.jctc.5b00935 5)

[5](https://doi.org/10.1021/acs.jctc.5b00935 5). Webb, B., & Sali, A. (2016). Comparative Protein Structure Modeling Using MODELLER. *Current Protocols in Bioinformatics*, *54*(1), 5.6.1-5.6.37. <https://doi.org/10.1002/cpbi.3>

6. Abraham, M. J., Murtola, T., Schulz, R., Páll, S., Smith, J. C., Hess, B., & Lindah, E. (2015). Gromacs: High performance molecular simulations through multi-level parallelism from laptops to supercomputers. *SoftwareX*, *1*–*2*, 19–25. https://doi.org/10.1016/j.softx.2015.06.001

7. Huang, J., Rauscher, S., Nawrocki, G., Ran, T., Feig, M., de Groot, B. L., Grubmüller, H., & MacKerell, A. D. (2017). CHARMM36m: an improved force field for folded and intrinsically disordered proteins. *Nature Methods*, *14*(1), 71–73. https://doi.org/10.1038/nmeth.4067

8. Smith, N. A., Wardak, A. Z., Cowan, A. D., Colman, P. M., Czabotar, P. E., & Smith, B. J. (2022). The Bak core dimer focuses triacylglycerides in the membrane. *Biophysical Journal*, *121*(3), 347–360. https://doi.org/10.1016/j.bpj.2021.12.043

9. Parrinello, M., & Rahman, A. (1981). Polymorphic transitions in single crystals: A new molecular dynamics method. *Journal of Applied Physics*, *52*(12), 7182–7190. https://doi.org/10.1063/1.328693

10. Essmann, U., Perera, L., Berkowitz, M. L., Darden, T., Lee, H., & Pedersen, L. G. (1995). A smooth particle mesh Ewald method. *The Journal of Chemical Physics*, *103*(19), 8577–8593. https://doi.org/10.1063/1.470117

11. Hess, B. (2008). P-LINCS: A parallel linear constraint solver for molecular simulation. *Journal of Chemical Theory and Computation*, *4*(1), 116–122. https://doi.org/10.1021/ct700200b
