# Supplementary material for: Key residues in the VDAC2-BAK complex can be targeted to modulate apoptosis
Source: PLoS Biol. 2024 May 2;22(5):e3002617. doi: 10.1371/journal.pbio.3002617 (PMC11098506; doi:10.1371/journal.pbio.3002617)
Supplement: Attachment — Submitted filename: Yuan_Response to reviewers.docx [file pbio.3002617.s010.docx]

We thank the reviewers for their considered and very positive comments on our manuscript.

Rev. 1:
The study done by Yuan et al. reports molecular analysis of the interaction between Bak and VDAC2, which constitute a key complex of the mitochondria-dependent apoptosis. Despite its significance in apoptosis regulation, this complex has been less investigated at an atomic level compared to the Bcl-2 family protein complexes, presumably because of technical difficulty. In this study, the authors attempted to address the molecular aspect of the Bak-VDAC2 intermolecular interaction, which were performed by a combination of deep scanning mutagenesis coupled with various biochemical approaches, including structural modeling, and cellular analyses. I consider that these results are interesting and scientifically important, and should substantially contribute to the precise understanding of Bak-mediated apoptosis. However, several points should be addressed before final decision, which are listed below.

>Page 5
- The authors described that the residues at the Bak hydrophobic groove, such as Arg88 and Tyr89, are proximal to the VDAC2 β10-11 loop, which was verified by cysteine cross-linking analysis. Figure 2B shows the location of those residues, but the hydrophobic groove is undiscerned at the structure of Bak. I suggest the authors to show it more clearly. Maybe surface representation of the Bak structure will be helpful.

We thank the reviewer for this suggestion. We now present the structure with an overlaid surface representation to better highlight the hydrophobic groove surface as a putative binding site for BH3-only domains and also VDAC2 loop.

>Page 6
- The authors also mentioned that the canonical hydrophobic groove of Bak is involved in its interaction with VDAC2. This groove is well known as the binding pocket of the BH3 domain from the Bcl-2 family proteins, implying the possibility of the β10-11 loop VDAC2 and the Bak-binding BH3 domains are competitive to associate with Bak. Therefore, I considered that the structural comparison analysis of Bak in a VDAC2-bound (modeled in this study; shown in Figure 3) and in BH3-bound forms (determined by numerous previous studies) should be added to this study.

We thank the reviewer for their comments. The modelled interaction of VDAC2 with the BAK hydrophobic groove is consistent with our cross-linking data. However, as we discussed in Figure 3 legend, this hypothetical model is one of several potential models, hence we believe a direct comparison with experimentally determined structures of BAK with bound BH3 peptides might be misleading.

>Page 8-9
- It is written that stabilisation of the BAK-VDAC2 complex interferes with apoptosis. But is it certain that the cells indeed underwent "apoptotic" death? The cell death analysis should be compensated by additional methods to confirm it, such as the caspase cleavage assay.

To remove any uncertainty as to the mode of cell death, we have used BH3-mimetic compounds that are known to specifically and potently trigger intrinsic apoptosis. We have now clarified this in the text and cited supported literature lines 263-264 (Oltersdorf et al. 2005 Nature, Kotschy et al 2016 Nature, Wang et al. 2020 ACS med Chem Lett.).

> Page 10
It is written that "we investigated cytochrome c release and cell death" with a delete line that should be removed.

Corrected.

> Page 10
The effects of WEHI-9625 were tested in MEFs expressing wild type VDAC2 or VDAC2A172W, but not in cells expressing VDAC2A172L. Is there any reason?

These experiments tested the hypothesis that the A172 VDAC2 mutation would limit the activity of small molecule BAK inhibitor WEHI-9625 that stabilises the BAK-VDAC2 interaction to inhibit apoptosis. As the A172L VDAC2 mutation itself stabilises interaction between VDAC2 and BAK, thereby constitutively inhibiting BAK activity, this mutant was not appropriate to test this hypothesis. Hence, we examined the VDAC2 A172W mutation as it did not inherently limit BAK proapoptotic activity. This enabled us to clearly show that WEHI-9625 no longer stabilises the BAK:VDAC2 interaction when VDAC2 A172W is expressed and allowed us to identify a potential binding site for WEHI-9625 on VDAC2.


Rev. 2: Tudor Moldoveanu – note that this reviewer has signed his review
This timely, well-executed, and well-presented study, based on an initial, elegant deep scanning mutagenesis experiment, reveals additional insights into the direct interactions between BAK (and BAX) and VDAC2, which regulate apoptotic response. This interaction could be targeted to inhibit (or activate) apoptosis as suggested by a mouse BAK selective compound WEHI-9625 which stabilizes the BAK-VDAC2 inhibitory complex, although the mechanism of this inhibitor is yet to be defined in future structural investigations as the authors suggest.

I have one question related to the use of MCL-1 and BCL-xL selective inhibitors to trigger apoptosis. Have the authors also used a more potent combination of S63845 and ABT-263 or ABT-737 to induce apoptosis and under those conditions is the VDAC2-mediated inhibition of BAK/BAX-induced apoptosis more readily alleviated between the differentVDAC2 A172 mutants? This information would be very informative for targeting the VDAC2/BAK/BAX inhibitory axis.

We thank the reviewer for this suggestion. When assessing the effect of VDAC2 A172 mutation on BAK apoptotic activity (i.e. in Bax-/- cells, Fig 5A) we used S63845 and A1131852 as potent stimulators of cell death given that MCL-1 and BCL-xL are the major repressors of BAK. Inhibition of either BCL-2 and BCL-w with ABT737 or ABT263 would not be expected to cause significantly greater cell death in these Bax-/- deficient cells. When assessing the effect on BAX (in BAK-/- cells, Fig 5C) or both BAX and BAK (in wild-type cells, Fig 5D), we instead employed ABT-737 in combination with S63845 to inhibit BCL-2 in addition accounting for the role BCL-2 in inhibiting BAX, but not BAK proapoptotic function. As proposed by the reviewer, and shown in Fig 5D, ABT-737 + S63845 did indeed reveal significant differential apoptosis with VDAC2 A172 mutation.

A couple of typos include:

Page 9 A113852 should probably by A1331852.

Corrected.
Page 10 The strikethrough should be deleted.
Corrected.


Rev. 3: Christoph Borner – note that this reviewer has signed his review
This is an impressive, nicely performed and convincing study further determining the interaction between VDAC2 and BAK and its consequence for BAK inhibition and activation. The authors show that A172 within the VDAC2 β10-11 loop interacts with the hydrophobic binding pocket of BAK. Obstructive labelling disrupts this interaction, A172 can be crosslinked to the BAK pocket, and mutations of A172 affect BAK localization, the stability of the VDAC2-BAK complex and the proapoptotic activity of BAK. It seems that the β10-11 loop structure of VDAC2 directly interacts with the BAK pocket rather than a helix structure as it is characteristic for the classical BH3-peptide/BCL-2 interaction. But as for Bcl-2 family interactions, BH3 proteins also seem to compete with VDAC2 for the BAK groove, thereby releasing BAK for oligomerization, cytochrome c release and apoptosis.

A major part of the study shows that the type of substitution made to VDAC2 A172 can have variable impact on the stability of the VDAC2-BAK complex. Mutations to medium sized hydrophobic sidechains like Ile, Leu, Cys stabilise the complex, whilst the mutation to a large hydrophobic residue like Trp is less able to restrain BAK. Interestingly, a previously developed compound WEHI-9625 seems to phenocopy the stabilizing effect of Leu or Ile mutations at A172 thus explaining why WEHI-9625 is an effective BAK inhibitor.

Since VDAC2 is also required for BAX localization to mitochondria and regulating its pro-apoptotic activity, the authors also studied the effect of the A172 mutants on BAX. As shown in Figure 4F, these mutants had opposite effects on BAX interaction. A172L/I/C VDAC2 mutations destabilized the VDAC2-BAX interaction while more BAX becomes associated with VDAC when A172 is mutated to Trp. However, in Bak-/-Vdac2-/- cells the VDAC2 A172L/I mutations still had an inhibitory effect on BAX leading to diminished apoptosis in response to BH3-mimetic treatment (Figure 5C). The authors propose that while BAK is less freed from VDAC2 A172L/I/C, BAX becomes more cytosolic, thereby exerting the same effect, namely less apoptosis induction.

The VDAC2-BAK interaction studies are convincing and provide sufficient novelty for a publication in PLOS Biology. However, the interpretation of the VDAC2-BAX data is less clear.

Minor point:
What happens with the BAX pool that binds less to VDAC2 L172L/I/C in Figure 4F? In contrast to what the authors suggest, it most likely remains on mitochondria rather than being retrotranslocated to the cytosol (no increase in cytosolic BAX in Figure 4G). But in what form is BAX present on mitochondria under these circumstances? One possibility is that it is responsible for the minor tBID-triggered cytochrome c release from Vdac2-/- mitochondria (Figure 4H) or the BH3-mimetic-triggered cell death in Bak-/-Vdac2-/- MEFs expressing the VDAC2 L172L/I/C mutant (Figure 5C).

We thank the reviewer for these insights. We do find that BAX association with mitochondria is consistently reduced upon disruption or destabilisation of the VDAC2 interaction due to A172 mutation, but that this effect is only observed in cells that also lack BAK (please see Fig S3C). This is consistent with our previous findings that either VDAC2 or BAK are required for BAX to efficiently localise to mitochondria (Ma et al CDD 2014). Hence, we conclude that BAX cytosolic redistribution/retrotranslocation does contribute to the observed reduction in BAX apoptotic activity in Bak-/- cells upon mutation of VDAC2 A172L/I as reported in Fig 5C.

The conformation of any resident mitochondrial BAX dissociated from VDAC2 is unclear and we cannot exclude that this population contributes to the cyt c release observed in Fig 4H. However, we note that these data in Fig. 4H are consistent with cytochrome c release observed in BAX-deficient cells (Fig 4I), supporting BAK as the predominant effector of cyt c release in Vdac2-/- cells in Fig 4H. We have now amended the text on page 8 lines 244-252 to better reflect these data:

*“To test if this stabilisation impacted BAK’s ability to mediate MOMP and cytochrome c release, supernatant and membrane fractions were fractionated following treatment of mitochondria‑enriched heavy membrane with tBID (Fig 4H) in cell lines expressing wildtype VDAC2 or VDAC2^A172I/L/W^. Cytochrome c release was delayed from mitochondria isolated from cells expressing, suggesting that a single point mutation on VDAC2 is sufficient to stabilise the BAK–VDAC2 complex and restrain BAK’s capacity to mediate MOMP (Fig 4H). As BAK is constitutively mitochondrial and BAX is predominantly cytosolic argues that BAK is likely the dominant effector of MOMP in these mitochondrial assays. Moreover, VDAC2^A172I/L^ mutation caused BAX to further dissociate from mitochondria, at least in cells lacking BAK (Fig S3C), consistent with our previous report that BAX requires either VDAC2 or BAK to efficiently target mitochondria [10]. However, we could not exclude that residual mitochondrial BAX contributed to cytochrome c release in these assays. Hence, to assess the impact of mutations to VDAC2^A172^ in the complete absence of BAX, we assessed cytochrome c release in Bax^-/-^Vdac2^-/-^ MEFs, reconstituted with VDAC2 variants, treated with BH3 mimetics (Fig 4I, Fig S3A, Fig S4). When BAK is the sole apoptosis effector, constraining mutations of VDAC2^A172I/L^ mutation inhibited cytochrome c release (Fig 4I).”*

In fact, diminished BH3-mimetic cell death due to VDAC2 L172L/I/C expression is more pronounced in Bax-/-Vdac2-/- (Figure 5A) than Bak-/-Vdac2-/- MEFs (Figure 5C). Why is this the case if the impact of the VDAC2 L172L/I/C mutants on BAX and BAK are the same?

The reviewer is correct that the diminished cell death due to VDAC2 A172L/I/C expression is more pronounced for BAK (Figure 5A) than it is for BAX (Figure 5C). This likely reflects the different effects of the A172L/I/C mutation on the BAK:VDAC2 and BAX:VDAC2 interactions; with BAK it stabilises, with BAX it destabilises. At the molecular level, we presume that the VDAC2 A172L/I mutation has a greater propensity to stabilise the interaction with BAK than dissociate BAX.

Can the authors show that in Vdac2-/- cells expressing the VDAC2 L172L/I/C mutants (Figure 4F), the BAX released from these mutant proteins oligomerizes (Blue native, crosslinking), especially upon treatment with BH3 mimetics (on cells or mitochondria in vitro)? Treating these cells or their isolated mitochondria with BH3 mimetics would not imply BAK because it is blocked by the strong VDAC2 L172L/I/C interaction and involve only BAX. I think this is an important experiment given the proposition of the authors to treat diseases caused by mitochondrial apoptosis with WEHI-9625. If WEHI-9625 only stabilizes the VDAC-2/BAK interaction and does not effectively retrotranslocate BAX to the cytosol, then BAX-mediated MOMP may still occur and the drug would not be as efficient as thought.

Our data (Fig 5D) show that in cells expressing both BAX and BAK, cell death is still limited by VDAC2 A172L/I mutation, so even if BAX is not fully retrotranslocated, its activity is still impaired. Moreover, that WEHI-9625 binds this site on VDAC2, supports that small molecule targeting of this region of VDAC2 might be a therapeutic opportunity to inhibit apoptosis. However, we agree with the reviewer that this may be dependent on context (e.g. relative levels of BAX/BAK, nature of the death stimulus) and so we have amended the Discussion text to reflect this on page 12 line 404 and page 13 lines 408-411.

*“That mutation to VDAC2^A172^ impacts both BAX and BAK activity suggests that this site of interaction is conserved for these two proteins. When VDAC2^A172^ is mutated to tryptophan it decreases the stability of the interaction with BAK, and more BAX becomes associated with VDAC2. Conversely, mutation of VDAC2^A172^ to Leu/Ile had opposing effects on VDAC2 interaction with these two proteins – destabilising interactions with BAX, but stabilising interactions with BAK. For BAX this results in reduced recruitment to the mitochondria, especially when BAK is absent, for BAK less freed protein on the mitochondria. In both scenarios there is a reduction of free BAX and/or BAK on the mitochondria available to induce MOMP, and therefore the ultimate consequence on cell death is the same. Excitingly, this suggests that this site on VDAC2 can be potentially targeted to inhibit apoptosis in cells expressing both BAK and BAX. Whilst this site on VDAC2 mediates interactions with both BAK and BAX, our data suggest that the effectiveness of targeting this site on VDAC2 to inhibit cell death will likely be more profound in cells, or with apoptotic stimuli, that predominantly rely on BAK.”*

In terms of the relevance to WEHI-9625 as a therapeutic agent, it is unclear how this compound specifically targets the mBAK-VDAC2 complex. One possibility is that it binds to a tertiary interface composed of a VDAC2 site (our work implicates the A172 region) and a mouse BAK site that is not conserved on human BAK or BAX. Our mutational analysis indicates that a compound targeting this site on VDAC2, but which is not dependent on a second interface on BAX or BAK could hold potential as a means to prevent mediation of apoptosis by either protein.

Rev. 4:

We thank the reviewer for their very positive comments.
In this study, Yuan and colleagues extensively define the functional relationship between VDAC2 and BAK (with some latter focus on BAX). Using the knowledge that disruption of VDAC2/BAK binding leads to degradation of BAK, they nicely couple this with deep-mutational scanning to identify putative VDAC2/BAK interacting residues. They then progress to validate and define these residues through cysteine and obstructive labelling of VDAC2, later defining a likely interaction between VDAC2 and BAK occurs via the canonical BAK hydrophobic groove. Finally, they demonstrate functional relevance of the VDAC2/BAK interacting residues, showing that stabilization of the interaction inhibits MOMP/cell death, whereas destabilization promotes BAK induced death.

In my view the study is timely, sheds significant light of the enigmatic relationship between VDAC2 and BAK activity in cell death, clearly has additional translational potential as well - serves as a rational starting point to either to inhibit or promote cell death. The authors' conclusions are fully supported by the data, and in my opinion the study should be accepted as is.
